# Improving the Health and Environmental Health Literacy of Professionals: Evaluating the Effect of a Virtual Intervention on Phthalate Environmental Health Literacy

**DOI:** 10.3390/ijerph21121571

**Published:** 2024-11-26

**Authors:** Kathryn S. Tomsho, Marlee R. Quinn, Zifan Wang, Emma V. Preston, Gary Adamkiewicz, Naima T. Joseph, Blair J. Wylie, Tamarra James-Todd

**Affiliations:** 1 Department of Environmental Health, Harvard T.H. Chan School of Public Health, Boston, MA 02115, USA; 2 Division of Maternal and Fetal Medicine, Department of Obstetrics and Gynecology, Boston Medical Center, Boston University School of Medicine, Boston, MA 02118, USA; 3 Division of Maternal and Fetal Medicine, Department of Obstetrics and Gynecology, Columbia University Irving Medical Center, New York, NY 10032, USA; 4 Department of Epidemiology, Harvard T.H. Chan School of Public Health, Boston, MA 02115, USA

**Keywords:** phthalates, reproductive health, environmental health literacy, intervention, clinical practice, obstetrics, environmental health

## Abstract

The American College of Obstetricians and Gynecologists provided updated guidance in 2021, recommending that reproductive health professionals should include discussion of environmental exposures with their patients. However, environmental health is seldom included in medical training, with endocrine-disrupting chemicals, such as phthalates—linked to adverse pregnancy outcomes—being among the least discussed. We developed a one-hour virtual educational intervention to train reproductive health professionals on the routes of phthalate exposure, potential associated health impacts, and suggestions on how to discuss exposure reduction with patients. The intervention was designed to include perspectives from patients, scientists, and clinicians. Using a pre/post/post design, we evaluated the impact of the intervention on reproductive health professionals’ phthalate-related reproductive health literacy via a validated environmental health literacy (EHL) scale, their confidence in discussing phthalates, and the frequency of discussions about phthalates with patients. All materials, including the study questionnaires and intervention materials, were administered virtually to reproductive health professionals (*n* = 203) currently seeing patients working in the United States. After completing the intervention, reproductive health professionals’ average EHL increased (pre-course: 22.3, post-course: 23.7, 2 months post-course: 24.0), as did their confidence in discussing phthalates with their patients (pre-course: 1% (2/203) reported being quite confident, post-course: 64% (131/203) reported being quite confident, and 2 months post course: 86% (174/203) reported being quite confident). Additionally, the reported frequency of discussions about phthalates with patients rose substantially (pre-course: 0% (0/203) reported usually discussing phthalates with patients, and 2 months post-course: 86% (175/203) reported usually discussing phthalates with patients): In line with the recommendations of the American College of Obstetricians and Gynecologists, this online phthalate educational intervention tool increased EHL among reproductive health professionals and shifted clinical care to include discussion about phthalates, a reproductive toxicant.

## 1. Introduction

In 2021, the American College of Obstetricians and Gynecologists (ACOG) provided updated guidance recommending the inclusion of discussions about environmental factors, including endocrine-disrupting chemicals (EDCs), as a part of standard clinical care. This guidance was informed by numerous studies linking EDCs to adverse reproductive health outcomes [1]. Among the most prevalent and concerning EDCs are phthalates. Exposure to this class of chemicals, commonly found in consumer products (such as deodorants, shampoos, or perfumes) [2,3,4] is associated with adverse pregnancy and gynecologic conditions, including preterm birth, hypertensive disorders of pregnancy, and uterine fibroids, all of which are associated with multigenerational morbidity and mortality [5,6,7,8]. Importantly, exposure to these non-persistent chemicals (which remain in the environment for brief periods after release) is modifiable through behavior modification [9]. Yet, standard obstetrics and gynecology training does not include education on phthalates or other EDCs [10,11].

As trusted health educators, clinicians play a key role in communicating important information about exposures that could impact health. However, to effectively convey such information, clinicians must possess sufficient knowledge and understanding of these exposures to share with their patients. Interestingly, most clinicians report that out of 10 common environmental exposures (i.e., mercury, lead, insecticides, pesticides, air pollution, volatile organic compounds, molds, polychlorinated biphenyls, bisphenols, and phthalates), they feel least confident discussing phthalates with their patients due to unfamiliarity with the subject matter [10].

Phthalates are a class of EDCs used in consumer products due to their utility as plasticizers, solvents, and lubricants [3,5,12,13]. Phthalates are often used as plasticizers to enhance the flexibility of plastics and as solvents and stabilizers in personal care products such as perfumes, hair oils, and lotions [3,12]. Phthalates are ubiquitous in modern life and can leach into the environment due to their non-covalent bonds [12]. Due to their global prevalence, exposure to phthalates is an international concern [14,15,16]. Primary routes of exposure to phthalates include ingestion, inhalation, and dermal absorption [12,13,17]. Exposure to phthalates has been associated with various health outcomes, including disruption of reproductive systems, cardiovascular impacts, and respiratory disease [5,6].

Perinatal exposures to phthalates have been associated with adverse health outcomes for both the parent and child [7,8,17,18,19,20,21,22,23,24]. Specifically, exposure to phthalates during pregnancy has been associated with adverse outcomes such as elevated blood pressure in mid-to-late gestation [20], excessive gestational weight gain [25], preterm birth [26,27], and reduced birthweight [28]. Furthermore, a pooled study of 16 United States cohorts examining exposure to phthalate mixtures found that higher urinary phthalate biomarker concentrations were associated with increased odds (ranging from 12% to 16%) of preterm birth per interquartile range increase [29].Additionally, hypothetical interventions to reduce perinatal phthalate exposure demonstrated substantial potential to decrease preterm birth rates, suggesting that “hypothetical interventions of 10% to 50% would correspond to an estimated mean of 2 to 12% reduction in preterm births” [29]. Thus, reducing perinatal phthalate exposure is advantageous for both parent and child health. The development of educational interventions to educate reproductive health professionals on the associations between phthalate exposures, sources of exposures, and health outcomes could contribute to a reduction in perinatal phthalate exposures and deliver tangible health benefits.

Despite the critical role clinicians play in educating their patients about potentially harmful environmental exposures, interventions aimed at improving clinician EHL remain limited. Addressing this gap is essential for meeting ACOG’s guidelines and improving clinical care for people of reproductive age. Such interventions could provide knowledge of environmental exposure risks and opportunities to exercise protective measures, improving health outcomes across multiple generations. Using a pre/post/post design, we evaluated the impact of an intervention on reproductive health professionals’ phthalate-related reproductive health literacy via a validated EHL scale, confidence in discussing phthalates, and the frequency of phthalate-related discussions with patients.

## 2. Materials and Methods

### 2.1. Study Design and Participant Recruitment

**Figure 1:**
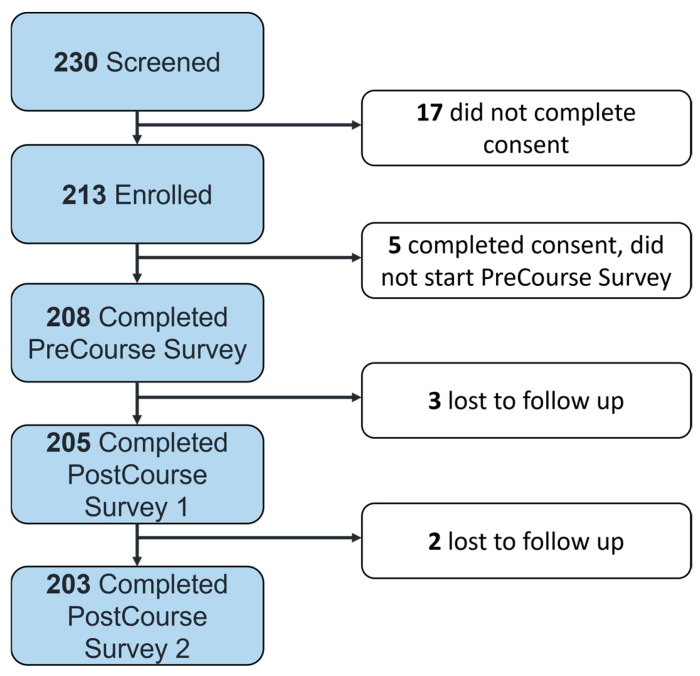
Flowchart of recruitment and retention in the IHEHLP Study.

For the Improving Health and Environmental Health Literacy of Professionals (IHEHLP) Study, we used a pre/post/post study design to assess whether a one-hour virtual educational intervention increased clinicians’ reproductive phthalate environmental health literacy. Participants were recruited virtually via the distribution of recruitment materials to professional networks for health professionals (e.g., obstetricians, gynecologists, family practitioners, nurses, etc.) in the United States between May 2023 and December 2023. Participants accessed the eligibility screening via hyperlinks to the eligibility screening material hosted on REDCap version 13.7.19, a secure online software designed for collecting data for research studies [30]. Participants were eligible to participate if they were healthcare providers with an active clinical practice that included reproductive healthcare. A total of 230 individuals were screened. All 230 who were screened met both eligibility criteria of being employed in a patient-facing role relevant to reproductive health and interacting with patients as part of their employment. Ultimately, 213 individuals consented to participate and enrolled, of whom 208 completed the pre-course survey, 205 completed the first post-course survey, and 203 completed the second post-course survey, leading to an approximately 95% retention rate. All follow-up activities for this study were completed by March 2024. Figure 1 illustrates the number of participants retained from the initial screening to the final survey in the IHEHLP Study.

### 2.2. Participant Questionnaires

Upon enrollment in the IHEHLP Study, participants were asked to complete the pre-course survey via REDCap. The survey included specific domains such as demographics, occupational and educational history, current discussion of environmental health topics in clinical care, the Phthalate Environmental Reproductive Health Literacy (PERHL) Scale [31], and a validated personal care product (PCP) questionnaire [32]. The PERHL Scale provides a numerical score for overall phthalate and reproductive EHL, ranging from 6 (low) to 30 (high). Additionally, it includes scores for six subscales, each ranging from 1 (low) to 5 (high): Awareness of Phthalate Reproductive Health Impacts, Scientific Uncertainty, Protective Behavior/Risk Control, Regulatory Interest, Awareness of Phthalate Exposure Routes, and General Phthalate Knowledge [31]. This scale was assessed for validity and reliability, as described previously [31].

Upon completion of the educational intervention, participants immediately completed the post-course survey via REDCap, which mirrored the pre-course survey. Two months after completing the first post-course survey, participants were invited via email to complete the second and final post-course survey, which included the same questions as the previous two surveys. Participants were compensated after completion of the pre-course survey and the final post-course survey.

### 2.3. Online Educational Intervention

The online educational intervention consisted of four videos, an interactive module, and two downloadable materials on phthalates. The objectives of the educational intervention were that, upon completion of the materials, participants would be able to (1) Identify common sources of exposure to phthalates, (2) Identify populations that are disproportionately exposed to phthalates, (3) Discuss possible phthalate exposures with their patients, and (4) Provide specific suggestions on how patients can reduce their exposures to phthalates.

The educational intervention began with two narrative-based patient perspective videos (approximately 2 min each). In the first video, a mother of a school-aged child described her experience of becoming aware of environmental chemical exposures, concerns about these exposures during pregnancy, desires for communication with health professionals regarding exposures and reproductive health, and implications of access to this information on her sense of agency and control. The second patient perspective video represents the perspective of a young woman who is considering becoming pregnant. She describes her experience as an immigrant and highlights differences in personal care products marketed to different ethnic groups in terms of ingredients and access to appropriately labelled products. She expresses her hope that clinicians will start discussing the ingredients in personal care products with their patients and their associated health impacts and available alternatives to reduce exposures.

After the patients’ perspective videos, participants watch a video from a scientist’s perspective (approximately 8 min). In this video, an environmental epidemiologist provides pertinent information regarding phthalates. Specifically, information is presented regarding sources of phthalate exposure and the social drivers of disparities in exposure to phthalates [32,33,34,35]. Additionally, the epidemiologist describes the health outcomes associated with exposure to phthalates and highlights populations who are at disproportionate risk for these outcomes, including certain racial and ethnic groups [25,36,37,38].

Upon completing the scientist’s perspective video, participants are directed to an interactive module developed by the Environmental Defense Fund (EDF). This interactive module outlines the existing regulations in the United States that pertain to ingredients in personal care products. They then engage in a series of interactive activities (matching and multiple choice) to reiterate information about regulations in personal care products. This interactive engagement module was included to achieve higher retention of information and improved long-term recall [39].

The final component of the virtual intervention is a video from the clinicians’ perspective (approximately 5 min). This video features two practicing obstetrician-gynecologists. They describe updated guidance from the ACOG, which recommends incorporating discussions about phthalates into regular reproductive healthcare [1]. Additionally, they outline the important role of reproductive health professionals in educating patients about environmental exposures as trusted health professionals. Finally, they suggest questions to ask patients to provide insight into potential phthalate exposures and suggestions for patients to reduce their phthalate exposures. The videos were developed in collaboration with obstetricians and gynecologists, who are a part of the first region Pediatric Environmental Health Specialty Unit, funded by the Environmental Protection Agency (EPA)/Agency for Toxic Substances and Disease Registry (ATSDR), to ensure that they were accessible and appropriate for inclusion in regular clinical practice. All videos were produced by EmVision, a media agency that creates videos at the intersection of science, storytelling, and social justice [40].

### 2.4. Data Analyses

Participant sociodemographic and occupational factors were assessed using descriptive statistics. Environmental health literacy was assessed by calculating the total PERHL Scale scores, as well as the score for each subscale (sum of the scores for every question within the subscale divided by the number of questions in that subscale). The overall PERHL score for each individual was calculated by summing the scores for the 6 subscales. Each participant was therefore assigned an overall PERHL score and 6 subscale scores. The mean scores for all participants were calculated for each time point: PreCourse before engaging in the intervention, PostCourse1 immediately after engaging in the intervention, and 2 Mo. Post Course two months after completing the PostCourse1 survey. Mean subscale scores for the sample population were also calculated for each time point.

Participants were asked to indicate their level of confidence in answering patients’ questions about phthalates at each of the three time points, with five response options: ‘Not at all Confident’, ‘Somewhat Confident’, ‘Moderately Confident’, ‘Quite Confident’, and ‘Very Confident’. The percentage of participants indicating each level of confidence was calculated per time point. Participants were additionally asked whether they currently discussed phthalates with their patients during regular clinical practice at each time point, with five response options: ‘Never’, ‘Occasionally’, ‘About Half the Time’, ‘Usually’, and ‘Always’. The percentage of participants indicating each level of frequency discussing phthalates with their patients was calculated per time point. Additionally, we stratified the results for levels of reported confidence and frequency of discussing phthalates with patients by occupation to assess whether there were differences by clinical role. For these stratified analyses, we collapsed the reported health professions into the following groups: doctors (Doctors of Medicine and Doctors of Osteopathic Medicine), nurses (Nurse Practitioners), and others (Physician Assistants and Others). We additionally performed stratified analyses to assess whether there were differences in impact based on occupation (doctors, nurses, or others) or the number of years in a position related to reproductive health (<5 years, 5−10 years, or more than 10 years).

All data were analyzed using R Software (version 2023.12.1, R Foundation for Statistical Computing). This intervention study was registered with clinicaltrials.gov (NCT06032143) and followed all ethical guidelines by the Harvard T.H. Chan Institutional Review Board.

## 3. Results

Table 1 contains the demographic information for the 203 participants in the IHEHLP Study. The median age of participants was 33 years, with a minimum age of 27 years and a maximum age of 41 years. In terms of racial and ethnic identification, 96 participants (47.3%) identified as White, 41 (20.2%) as Black/African American, 29 (14.3%) as East Asian, 29 (14.3%) as South Asian, 6 (3.1%) as Native Hawaiian/Pacific Islander, 1 (0.5%) as Mexican/Mexican American and 1 (0.5%) as Puerto Rican. A variety of occupational titles were represented, with 101 participants (49.8%) being Nurse Practitioners (NPs), 55 (27.1%) being Physician Assistants (PA-C), 46 (22.7%) were Doctors of Medicine (MD), 46 (22.7%) selecting ‘Other’, and 1 (0.5%) being Doctor of Osteopathic Medicine (DO). Participants generally had recent experience in positions relevant to reproductive health, with ninety-eight (48.3%) reporting 1−5 years, ninety-six (47.3%) reporting 5−10 years, and nine (4.4%) reporting 10−15 years. Finally, none of the participants had previous experience with environmental health, with two-hundred-one (99.0%) reporting ‘No Experience’ and two (1.0%) providing no response. Of the 213 participants enrolled in the IHEHLP Study, 208 completed the pre-survey, and 203 completed the intervention and subsequent two post-survey, leading to a 95% retention rate.

**Figure 2:**
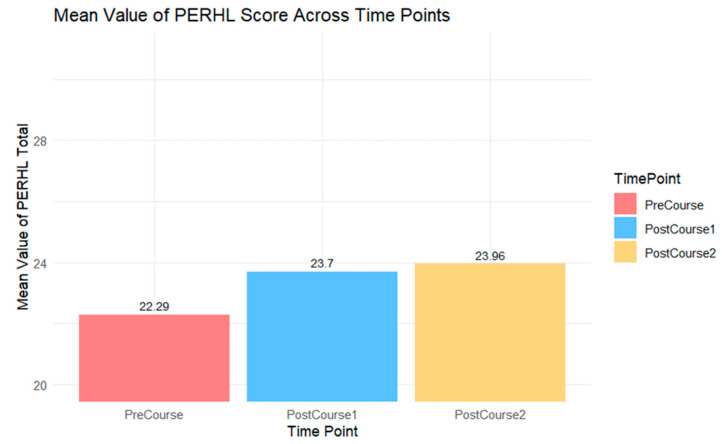
Mean PERHL scores across IHEHLP Study time points.

Overall, PERHL Scale scores increased from before engaging in the course (mean score 22.3 ± 1.44) to immediately after engaging in the course (mean score 23.7 ± 0.93) and again increased 2 months later (mean score 24.0 ± 0.94), as shown in Figure 2. Scores on the six subscale scores also generally increased after engaging in the educational intervention. Specifically, scores increased from the PreCourse survey to the second post-course survey (PostCourse2) for the following subscales: Awareness of Phthalate Reproductive Health Impacts (a 12.5% increase in mean score), Awareness of Phthalate Exposure Pathways (a 5% increase in mean score), Protective Behavior/Risk Control (a 10% increase in mean score), Regulatory Interest (a 5% increase in mean score), and General Phthalate Knowledge (a 10% increase in mean score). The mean scores for Scientific Uncertainty held constant at 2.1 through all three time points.

**Figure 3:**
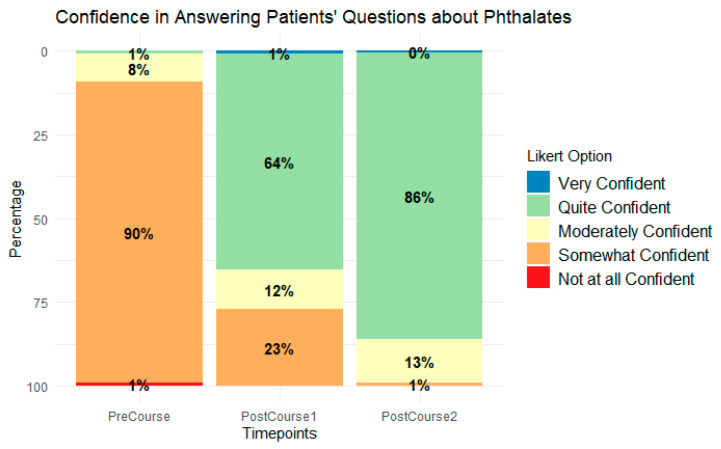
Reproductive health professionals’ confidence in answering patients’ questions about phthalates.

Participants’ confidence in discussing phthalates with their patients increased substantially after engaging in the educational intervention, as shown in Figure 3. Before engaging in the intervention (PreCourse), 1% reported being quite confident. Immediately after the intervention (PostCourse1), 64% reported being quite confident, and 2 months post-course (PostCourse 2), 86% reported being quite confident in answering patients’ questions about phthalates. Before the intervention (PreCourse), 0% reported usually discussing phthalates with patients. Two months after the intervention (2 Mo. Post Course), 86% reported usually discussing phthalates with patients.

**Figure 4:**
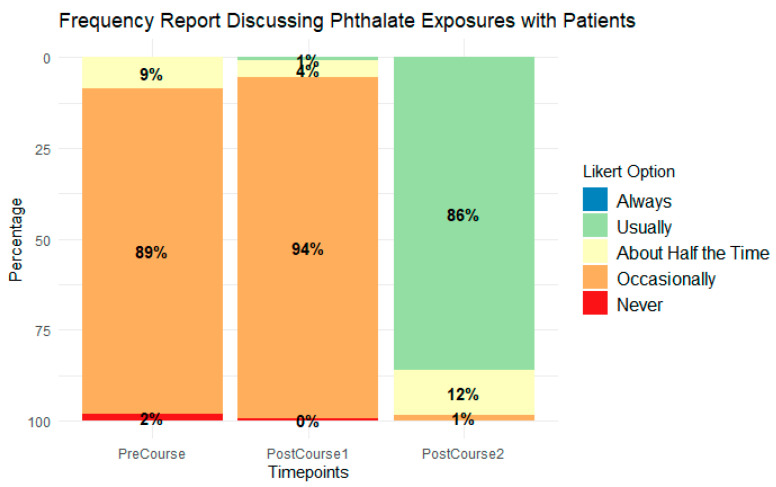
Self-reported reproductive health professionals’ frequency of discussing phthalates with patients.

Finally, as shown in Figure 4, participants reported a significant shift in the frequency with which they discussed phthalates with patients after engaging in this intervention. Before the intervention (PreCourse), 0% reported usually discussing phthalates with patients. Two months after the intervention (PostCourse2), 86% reported usually discussing phthalates with patients.

We found that the trends in changes to confidence in discussing phthalates with patients and the frequency of discussing phthalates with patients were similar across the three occupational groups (doctors, nurses, and others) and by number of years in a position relevant to reproductive health across the three study surveys (please see Supplementary Figures S1–S6).

## 4. Discussion

In this study, we presented an online, one-hour educational intervention in response to the updated guidance from ACOG to include discussions of environmental exposures like phthalates in reproductive healthcare. We found that the intervention significantly increased phthalate environmental health literacy among reproductive health professionals, as well as their confidence in and frequency of discussing phthalates with patients 2 months post-intervention. This scalable, accessible intervention holds the potential for educating reproductive health professionals to provide educational information on phthalate exposures, which could potentially improve reproductive health.

Pregnancy is a critical window of exposure to endocrine-disrupting chemicals due to the physiological processes involved and the ongoing development of the fetus [41]. Disruptions to development during this period can affect gene expression, leading to epigenetic impacts that can span generations, altered neurodevelopment, and reproductive health impacts [41,42]. Phthalates, specifically, have been associated with a range of health impacts for both the parent and child throughout the life course, and fetal exposure has been associated with alterations in the epigenome, preterm birth, and low birth weight [7,12,21,41].

Reproductive health professionals have a profound opportunity to provide information to pregnant individuals to facilitate a better understanding of phthalate exposures during pregnancy that can facilitate informed decision-making about reducing exposures for the pregnant individual [1,41].

A previous survey of ACOG fellows found that phthalates were the environmental exposure least discussed with patients in relation to adverse reproductive health outcomes (only 5% of fellows reported routinely discussing phthalates as part of prenatal care), despite their inclusion in the updated ACOG guidance for clinical care [10].Thus, a substantial gap exists between the call for inclusion of this topic in reproductive healthcare and health professionals’ preparedness to incorporate discussions of phthalates into clinical care. Increasing reproductive health professionals’ awareness and knowledge of phthalates is a critical step in addressing this gap. Additionally, previous studies have identified low confidence in discussing environmental exposures as a barrier to their inclusion in clinical care [10]. Increasing health professionals’ confidence in discussing environmental exposures is a second critical component of building their capacity to include this content in regular care. As a result of this educational intervention, there was a significant shift in the number of participating reproductive health professionals who reported usually discussing phthalates with their patients. This demonstrates that the educational intervention is not only effective in increasing EHL but can also lead to meaningful change in clinical practice by increasing the frequency with which reproductive health professionals discuss phthalates and phthalate avoidance strategies with their patients.

Despite repeated calls to include environmental health content in formal medical training, there has been little progress in doing so [43,44,45]. Notwithstanding the potential to provide structural support for environmental health via the training of medical practitioners, barriers persist, such as limited space in curricula, limited faculty capacity, and limited evidence that the implementation of environmental education will affect change in health outcomes [46]. Indeed, environmental medicine is broad and encompasses a range of different and continuously emerging contaminants to which individuals may be exposed. This poses substantial challenges to the development of curricula for training medical professionals on various potential exposures, particularly in the context of potential scientific uncertainty. Due to these barriers, several focused clinical and educational interventions have been tested for their potential to reduce environmental exposures. One study explored whether an interactive computer kiosk could enhance environmental education (addressing food, home, outdoor, and work environments) for low-income Latina prenatal patients. The authors found that participants reported learning something new and that the kiosk was an acceptable format for providing that information to the intended audience [47]. Another study assessed the effectiveness of the National Environmental Education Foundation’s Children’s Environmental Health Faculty Championship Initiative. Through this effort, the authors assessed whether a train-the-trainer model was effective in integrating pediatric environmental health competencies into medical and nursing practice. They found that there was a significant increase and retention of environmental health knowledge among 1559 healthcare professionals [48]. This effort demonstrated the efficacy of the developed program and the train-the-trainer model for incorporating environmental health content into medical education [48]. The results described here contribute to a growing body of research seeking to identify solutions to the gap in medical education regarding the inclusion of environmental health content. In addition to increasing knowledge and other measures of EHL as it relates to phthalates and health, our online intervention significantly increased reproductive health professionals’ confidence in discussing phthalates with their patients. This improved confidence continued through 2 months after engaging in the intervention, demonstrating its sustained impact. The sustained impact of the intervention in discussing phthalates with patients is crucial for equity, as it ensures that individuals from all backgrounds have access to informed conversations about potentially harmful environmental exposures, thus promoting equitable healthcare outcomes. Additional work should assess whether this confidence is maintained over longer periods of time after engaging with the educational intervention.

This intervention has several limitations. First, this study had a small sample size (*n* = 203). However, the original intention was to recruit 100 reproductive health professionals to achieve the desired statistical power, and this number was more than doubled due to overwhelming interest in the educational intervention. This enthusiasm surrounding the course demonstrates the interest and need for this type of brief and impactful training. Additionally, this study was designed to assess only the impact on reproductive health professionals’ phthalate environmental health literacy and the confidence of clinicians in communicating this information to patients. Additional research should assess the effect of this training on the patients of reproductive health professionals who have engaged in this educational intervention to assess whether it can also improve patients’ phthalate and reproductive environmental health literacy, reduce their exposure to phthalates, and prevent subsequent reproductive health impacts.

Environmental exposures to EDCs, such as phthalates, are linked to social determinants of health, with marginalized communities often facing disproportionate risks due to systemic inequities [33,49,50,51]. This educational intervention equips reproductive health professionals to address these exposures in clinical care. However, broader environmental and social factors may limit its generalizability. For example, patients’ ability to act on the information provided may be constrained by socioeconomic or structural barriers [52,53,54]. To maximize impact, integrating EHL into clinical practice should be complemented by policy interventions addressing systemic environmental injustices, thereby addressing disparities at both individual and population levels.

## 5. Conclusions

Overall, this educational intervention can assist with meeting the recommendations outlined by the ACOG in 2021. Specifically, it helps train reproductive healthcare professionals to be knowledgeable about phthalates and to discuss phthalate exposure reduction with their patients for possible improved reproductive health outcomes. Additional work should assess the best approach for implementing this educational intervention in various reproductive health settings, such as through formal continuing education programs. Further, there should be an assessment of the long-term sustainability of the intervention, as well as the dissemination of the information within the training, to ensure that it is provided in an equitable manner to reproductive health professionals and their patients, preventing the perpetuation of existing exposure and health disparities.

## Figures and Tables

**Table 1:** Demographic characteristics of IHEHLP Study participants (*n* = 203).

Characteristics	All (*n* = 203)
	Median [Min, Max] or *n* (%)
**Age at Enrollment (years)**	33.0 [27.0, 41.0]
**Race**	
Black/African American	41 (20.2%)
East Asian	29 (14.3%)
Mexican/Mexican American	1 (0.5%)
Native Hawaiian/PI	6 (3.0%)
Puerto Rican	1 (0.5%)
South Asian	29 (14.3%)
White	96 (47.3%)
**Occupational Title**	
Doctor of Medicine (MD)	46 (22.7%)
Doctor of Osteopathic Medicine (DO)	1 (0.5%)
Nurse Practitioner (NP)	101 (49.8%)
Physician Assistant (PA-C)	55 (27.1%)
Other	46 (22.7%)
**Years Employed in Position Relevant to Reproductive Health**	
1−5 years	98 (48.3%)
5−10 years	96 (47.3%)
10−15 years	9 (4.4%)
**Previous Experience with Environmental Health**	
No	201 (99.0%)
No Response	2 (1.0%)

## Data Availability

The raw data supporting the conclusions of this article will be made available by the authors upon request.

## Supplementary Materials

The following supporting information can be downloaded at https://www.mdpi.com/article/10.3390/ijerph21121571/s1, Figure S1: Distribution of Reproductive Health Professionals’ Overall PERHL EHL Scores at Pre/Post1/Post2 Timepoint, Stratified by Occupation; Figure S2: Proportion of Reproductive Health Professionals; Confidence Discussing Phthalates with Patients at Pre/Post1/Post2 Timepoints, Stratified by Occupation; Figure S3: Proportion of Frequency Reproductive Health Professionals Report Discussing Phthalates with Patients at Pre/Post1/Post2 Timepoints, Stratified by Occupation; Figure S4: Distribution of Reproductive Health Professionals’ Overall PERHL EHL Scores at Pre/Post1/Post2 Timepoints, Stratified by Years in Occupation; Figure S5: Proportion of Reproductive Health Professionals’ Confidence Discussing Phthalates with Patients at Pre/Post1/Post2 Timepoints Stratified by Years in Occupation; Figure S6: Proportion of Frequency of Reproductive Health Professionals Report Discussing Phthalates with Patients at Pre/Post1/Post2 Timepoints, Stratified by Years in Occupation.
