# Supplementary material for: Improving the Health and Environmental Health Literacy of Professionals: Evaluating the Effect of a Virtual Intervention on Phthalate Environmental Health Literacy
Source: Int J Environ Res Public Health. 2024 Nov 26;21(12):1571. doi: 10.3390/ijerph21121571 (PMC11675889; doi:10.3390/ijerph21121571)

Supplementary Materials

Figure S1: Distribution of Reproductive Health Professionals' Overall PERHL EHL Scores at Pre/Post1/Post2 Timepoints, Stratified by Occupation

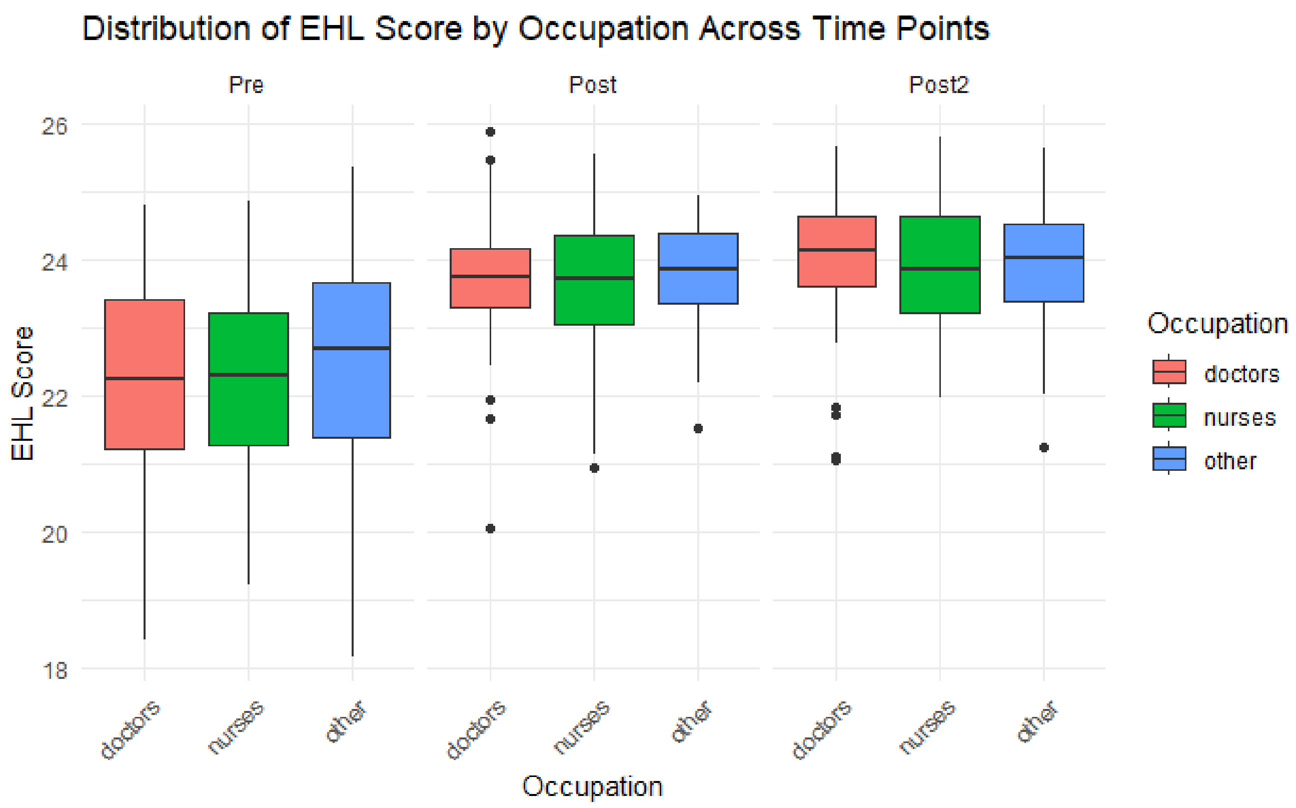

Figure S2: Proportion of Reproductive Health Professionals' Confidence Discussing Phthalates with Patients at Pre/Post1/Post2 Timepoints, Stratified by Occupation

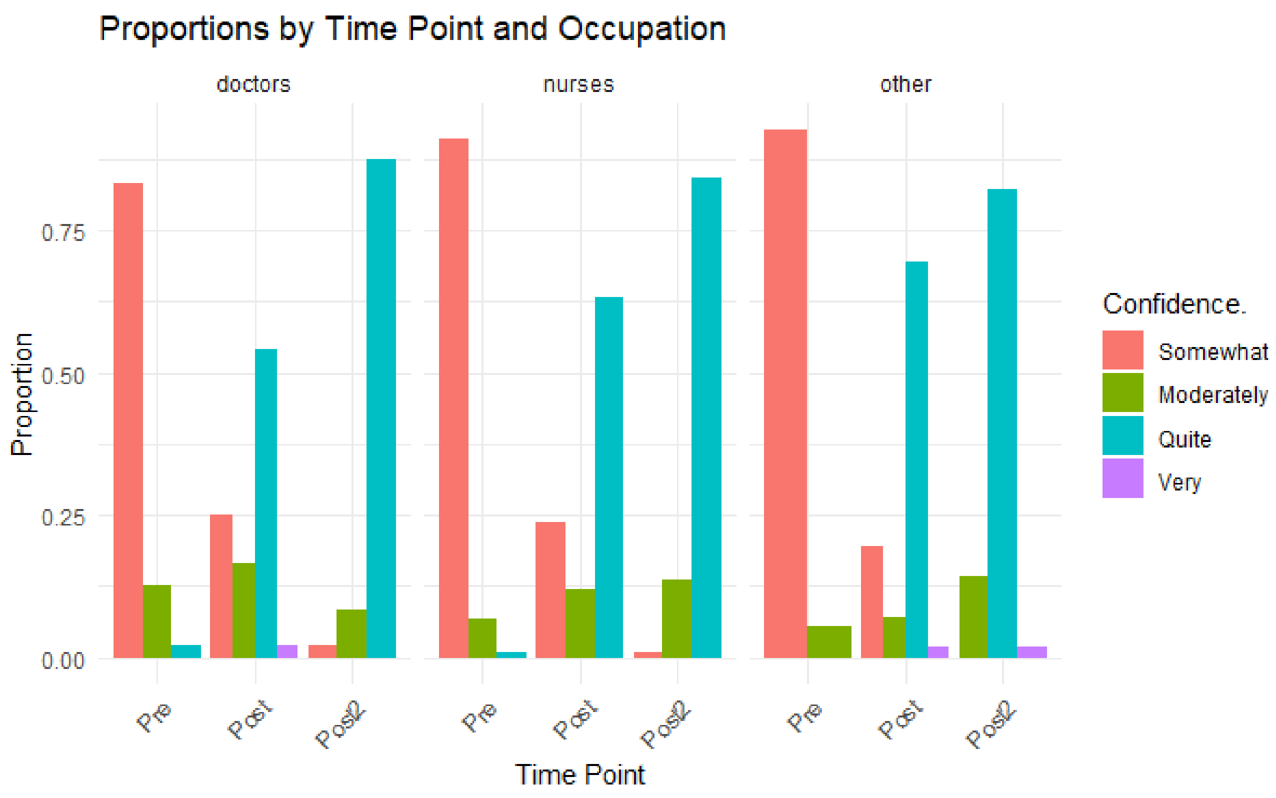

Figure S3: Proportion of Frequency Reproductive Health Professionals Report Discussing Phthalates with Patients at Pre/Post1/Post2 Timepoints, Stratified by Occupation

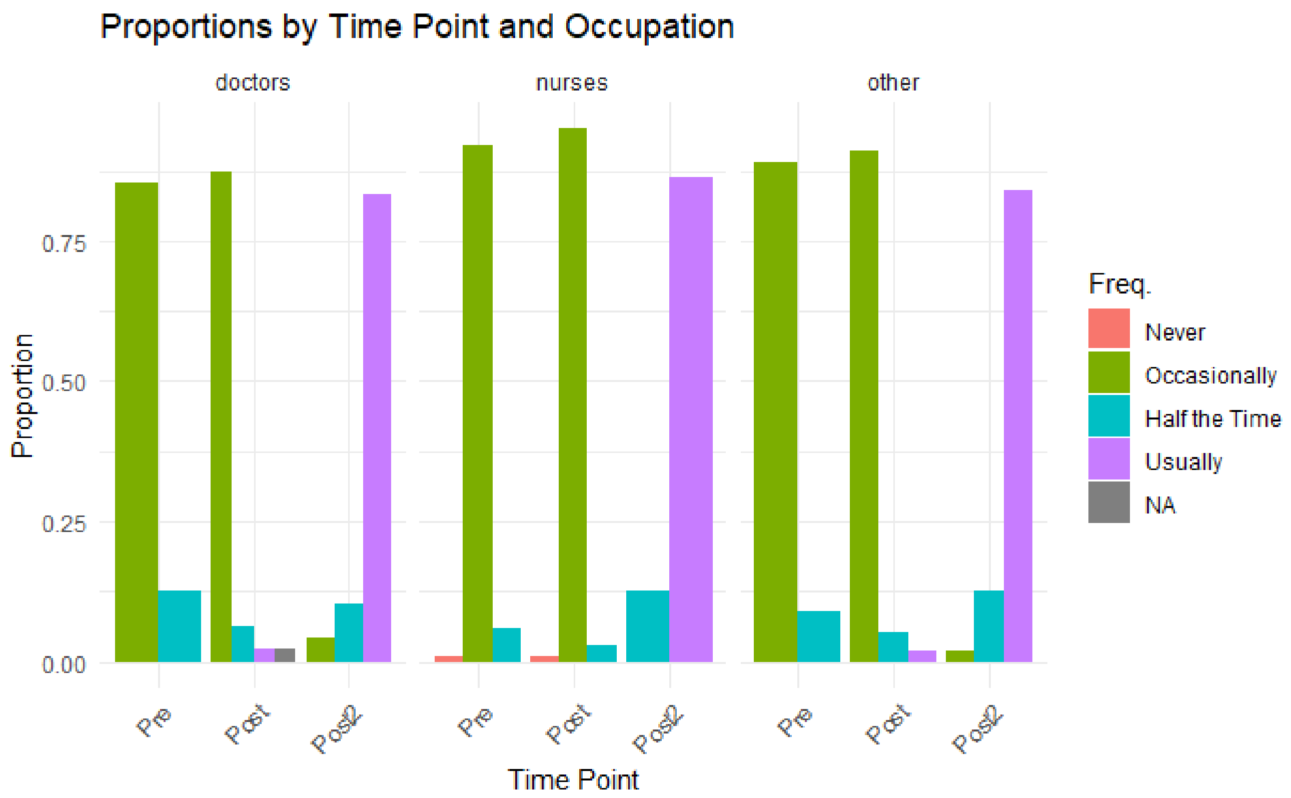

Figure S4: Distribution of Reproductive Health Professionals' Overall PERHL EHL Scores at Pre/Post1/Post2 Timepoints, Stratified by Years in Occupation

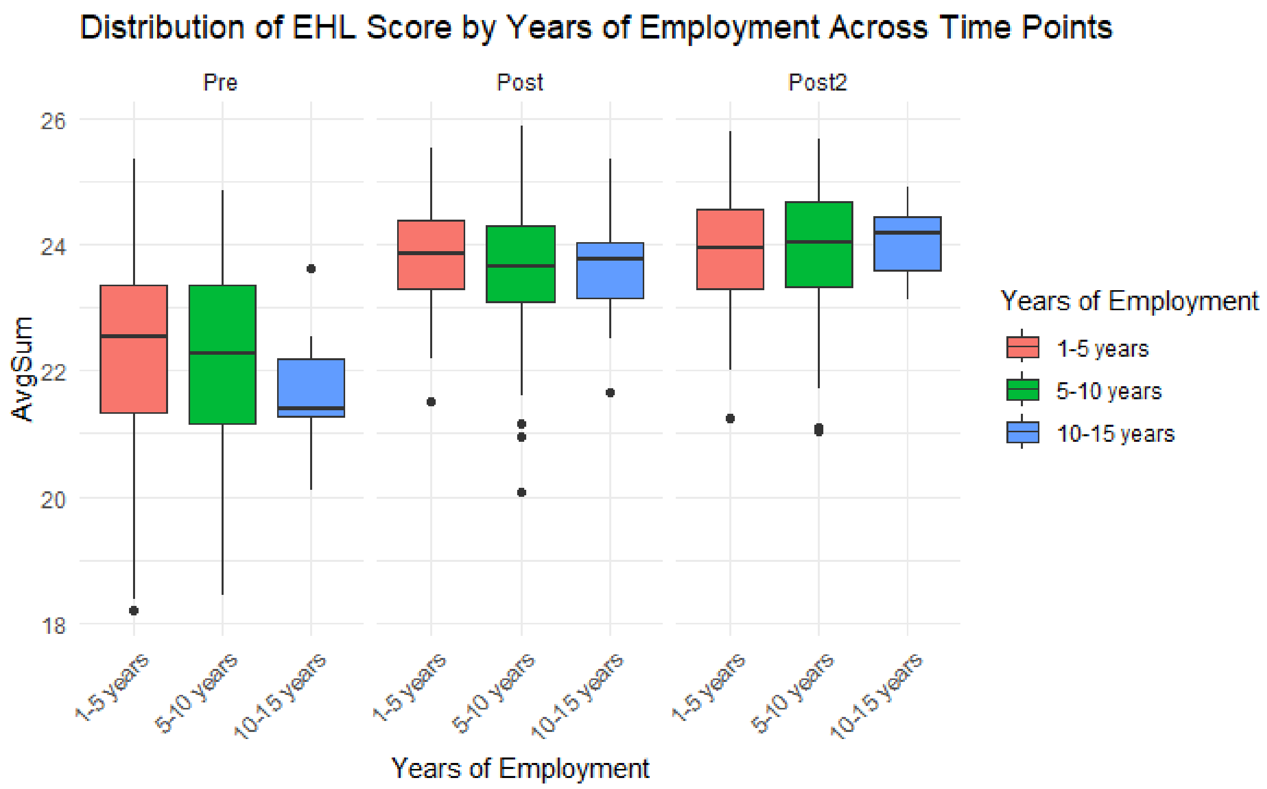

Figure S5: Proportion of Reproductive Health Professionals' Confidence Discussing Phthalates with Patients at Pre/Post1/Post2 Timepoints, Stratified by Years in Occupation

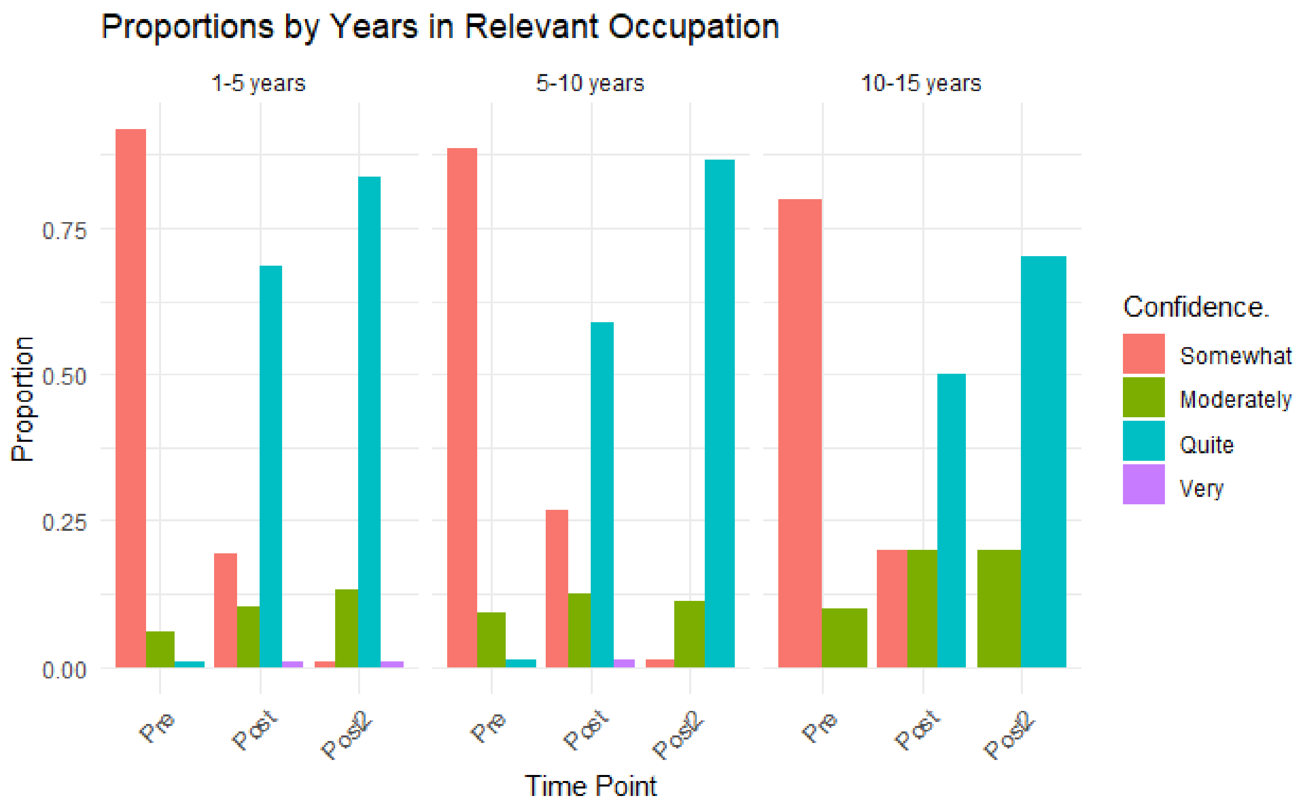

Figure S6: Proportion of Frequency Reproductive Health Professionals Report Discussing Phthalates with Patients at Pre/Post1/Post2 Timepoints, Stratified by Years in Occupation

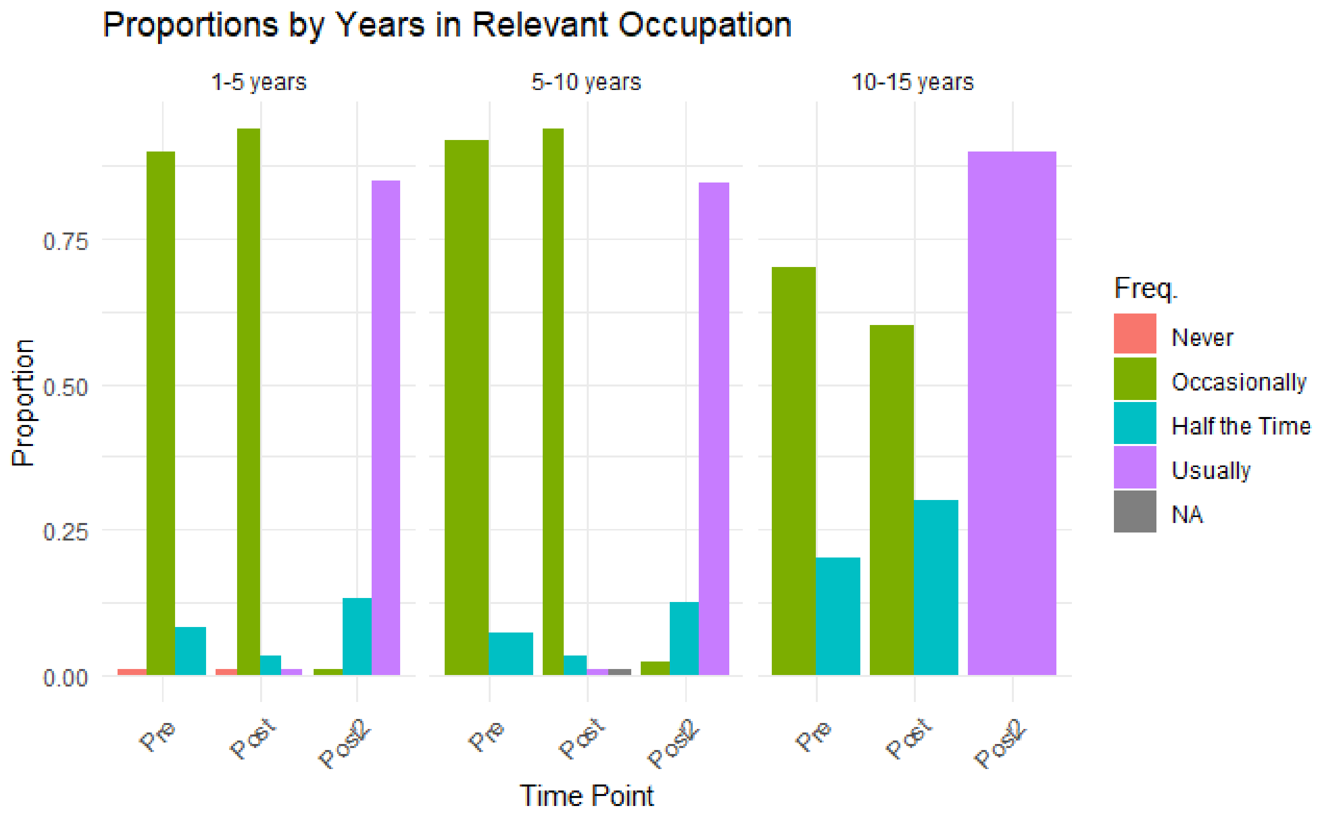

Supplement: Supplementary file 1 [file ijerph-21-01571-s001.zip › ijerph-3226018-supplementary.pdf]
